# Comparative Structural Analysis of Escherichia Coli Cyay at Room and Cryogenic Temperatures Using Macromolecular and Serial Crystallography

**DOI:** 10.1002/cbic.202500442

**Published:** 2025-10-28

**Authors:** Alaleh Shafiei, Nilufer Baldir, Jongbum Na, Jin Hae Kim, Hasan DeMirci

**Affiliations:** ^1^ Department of Molecular Biology and Genetics Koc University 34450 Istanbul Turkey; ^2^ Department of New Biology Daegu Gyeongbuk Institute of Science and Technology (DGIST) Daegu 42988 Republic of Korea; ^3^ SLAC National Laboratory Stanford PULSE Institute Menlo Park 94025 CA USA; ^4^ Koc University Isbank Center for Infectious Diseases (KUISCID) Koc University 34010 Istanbul Turkey

**Keywords:** cyaY, frataxin, Friedreich ataxia, room‐temperature crystallography, structural biology

## Abstract

Frataxin is a 23 kDa mitochondrial iron‐binding protein involved in the biogenesis of iron–sulfur (Fe–S) clusters. Deficiency in frataxin is associated with Friedreich's ataxia, a progressive neurodegenerative disorder. CyaY, the bacterial ortholog of eukaryotic frataxin, is believed to function as an iron donor in Fe–S cluster assembly, making it a key target for structural and functional studies. In this work, a comprehensive structural analysis of the *Escherichia coli* CyaY protein is presented, comparing its structure at room temperature and cryogenic conditions. Notably, the first room‐temperature structures are obtained using the Turkish Light Source “Turkish DeLight” X‐ray diffractometer and serial synchrotron X‐ray crystallography, marking a significant step forward in understanding CyaY under near‐physiological conditions.

## Graphical abstract

Room‐temperature (RT) crystallography of *E. coli* CyaY, the bacterial frataxin homolog, reveals subtle but functionally relevant differences from cryogenic structures. The conserved fold shows distinct side‐chain conformations and water networks, highlighting dynamics often hidden at low temperatures. Reduced radiation damage makes RT structures more suitable for accurate modeling and structure‐based drug design.

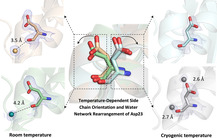

## 1 Introduction

Iron–sulfur clusters (ISCs) have been identified as one of the most ancient redox‐active co‐factors in the cell.^[^ ^1^ ^]^ These clusters play a critical role in a diverse range of biological processes, including electron transport, gene expression, gene regulation, photosynthesis, nitrogen fixation, enzyme‐substrate binding, DNA repair and replication, and RNA modification.^[^ ^2^, ^3^, ^4^, ^5^ ^–^ ^6^ ^]^ ISCs assembly occurs through conserved systems found across prokaryotes, archaea, and eukaryotes.^[^ ^7^ ^]^ These systems provide Fe–S clusters to a wide range of apo‐proteins.^[^ ^8^ ^,^ ^9^ ^]^

The *Escherichia coli*ISC system is the most well‐studied model system because of its lower complexity and since it encodes several protein subunits that have eukaryotic homologs. Cluster formation needs the intricate interactions and interplays of these multi‐proteins and other accessory proteins whose roles are not well understood. One such partner is the frataxin protein. In eukaryotes, frataxin deficiency results in loss of the activity of the ISC enzyme desulfurase, causing iron accumulation, and increased sensitivity to oxidative stress.^[^ ^10^ ^,^ ^11^ ^]^ In humans, reduced levels of frataxin expression are closely linked to Friedreich's ataxia, an autosomal recessive neurodegenerative disease.^[^ ^12^ ^,^ ^13^ ^]^ Frataxin has been proposed to function as an iron‐donor, an iron‐chaperone, or an iron sensor regulator of the ISC system.^[^ ^14^ ^–^ ^16^ ^]^ CyaY is of interest to researchers as it is the bacterial ortholog of eukaryotic mitochondrial frataxin.^[^ ^12^ ^,^ ^13^ ^]^ Moreover, it exhibits remarkable evolutionary conservation from bacteria to higher eukaryotes^[^ ^12^ ^]^ and shares 25% sequence identity with human and yeast frataxin (FXN/Yfh1).^[^ ^13^ ^]^

Prior crystallographic structural studies on CyaY and other proteins from the biogenesis of ISCs have been conducted at cryogenic temperatures.^[^ ^14^ ^–^ ^17^ ^]^ Despite the insights they provided, as a consequence of the fact that proteins freeze in the intermediate timescales while cryocooling,^[^ ^18^ ^]^ lowered temperatures have the potential to limit the identification of alternative protein conformations that might be functionally important.^[^ ^19^ ^,^ ^20^ ^]^ In addition, they have failed to show structural and functional heterogeneity of the complexes involved in cluster biogenesis. Room temperature crystal structures capture natural protein movements that are essential for understanding how proteins function. In one study, two methods—electron density over noise distributions (END) and real‐space atomic positioning with inherent data (RAPID)—were introduced to improve the detection of these dynamics. END enhances electron density maps by adjusting for local noise, while RAPID estimates how precisely each atom is positioned. Together, they reveal flexible or hidden conformations that are often missed when structures are determined at cryogenic temperatures.^[^ ^21^ ^]^

Traditional protein models often depict a single, static structure—but in reality, proteins are dynamic. They shift, wiggle, and adopt multiple conformations, especially in flexible regions like side chains and loops. Room‐temperature crystallography captures these natural motions more accurately than cryogenic methods. Tools like qFit take advantage of this by analyzing electron density maps to model multiple conformations simultaneously. These multi conformer models reveal hidden flexibility and provide deeper insights into protein dynamics and function, offering a more realistic view of how proteins behave in their native environments.^[^ ^22^ ^]^

Room‐temperature crystallography techniques, including serial crystallography, are advancing rapidly.^[^ ^23^ ^]^ Room‐temperature crystal structures are being used more frequently in computational simulations.^[^ ^24^ ^,^ ^25^ ^]^ Serial crystallography is now widely used to gain structural and dynamic insights, particularly in time‐resolved studies and X‐ray free‐electron laser (XFEL) experiments.^[^ ^26^ ^]^ It also supports structure‐based drug design^[^ ^27^ ^]^ and has the key advantage of not requiring large crystals.

To address the potential limitations of existing cryogenic protein structures in studying iron sulfur cluster biogenesis, we determined the first room temperature X‐ray crystal structure of CyaY from *E. coli* (referred to as RT^XRD^). This structure was obtained through *serially* data collection from multiple crystals using our Turkish DeLight home X‐ray source to reduce radiation damage.^[^ ^28^ ^,^ ^29^ ^]^ Additionally, we acquired the first room‐temperature serial synchrotron X‐ray crystallograph**y** (SSX) structure of CyaY (referred to as RT^ssk^) using a fixed‐target approach at the P14 EMBL Beamline in Hamburg. This method enables data collection at room temperature while minimizing radiation damage by distributing partial datasets across numerous micro crystals, providing high‐quality oscillation data and preserving protein integrity.^[^ ^30^ ^]^ Furthermore, we obtained two cryogenic structures of CyaY (referred to as Cryo^XRD^ and Cryo^MX^) using single crystals at the Turkish DeLight home X‐ray source and Diamond Light Source, respectively. For a rigorous comparison, all structures were derived from crystals grown under identical crystallization conditions.

The room‐temperature CyaY structures may serve as a foundation for further studies to elucidate the mechanism of ISC assembly at near physiological temperatures. Given that ISC biogenesis is a highly dynamic process requiring both iron and oxygen, time‐resolved studies are crucial to understanding how clusters fully form on protein complexes in real time. These reactions are best observed within room‐temperature crystals, as the process might be sensitive to cryogenic conditions, which can obscure intermediate states and disrupt the natural progression of cluster formation. This SSX structure provides a solid starting point for advancing time‐resolved serial synchrotron crystallography, which will deepen our understanding of the mechanisms underlying cluster assembly. Importantly, as CyaY is the bacterial ortholog of human frataxin—implicated in Friedreich's ataxia—these insights may also inform structure‐based drug design strategies aimed at targeting frataxin‐related dysfunction.

## 2 Results and Discussions

### 2.1 Structural Insights from Temperature‐Dependent Comparative Analysis of CyaY

#### 2.1.1 First Room‐Temperature Structure of CyaY Revealed by X‐Ray Diffraction and Serial Synchrotron Crystallography

We report the first ambient‐temperature X‐ray crystal structure of the CyaY protein (RT^XRD^) in its monomeric form, determined at a resolution of 2.0 Å using the Turkish DeLight X‐ray diffractometer (**Table** **1**). In addition, we determined the first room‐temperature SSX dataset for CyaY (RT^SSX^), collected from microcrystals at the P14 beamline operated by EMBL at PETRA III, DESY (Hamburg, Germany), which yielded a structure at 1.7 Å resolution (Table 1). For comparison, cryogenic datasets were also collected at the Turkish DeLight source (Cryo^XRD^) and at the Diamond Light Source (Cryo^MX^), achieving higher resolutions of 1.5 and 1.06 Å, respectively (Table 1). All crystals were obtained under the same crystallization conditions in a buffer containing 0.1 M citric acid and 4.0 M sodium chloride (pH 4.0), enabling a more detailed analysis of the protein's structure. Data collection statistics are summarized in Table 1. CyaY structure comprises 106 residues consisting of 3 α‐helices and 6 *β*‐strands. The topology in our structures remains unchanged from the previously reported CyaY structures.^[^ ^15^ ^,^ ^31^ ^]^

**Table 1:** Data collection and refinement statistics.

Datasets	RT^XRD^	Cryo^XRD^	Cryo^MX^	RT^SSX^
PDB IDs	8HZ1	8IVK	9V62	9 V63
				
Data Collection temperature	298	100	100	293
Data collection	–	–	–	–
X‐ray source	Turkish DeLight	Turkish DeLight	Diamond	Petra III
Space group	P 3_2_21	P 3_2_21	P 3_2_21	P 3_2_21
Cell dimensions				
*a*, *b*, *c* [Å]	45.166, 45.166, 101.129	44.456, 44.456, 98.425	44.48, 44.48, 98.16	45.20, 45.20, 101.10
*α*, *β*, *γ* [°]	90, 90, 120	90, 90, 120	90, 90, 120	90, 90, 120
Resolution [Å]	101.08–2.00(2.07–2.00)	20.73–1.50(1.55–1.50)	98.17–1.06(1.08–1.06)	39.14–1.7(1.83–1.7)
*CC* (½)	0.877(0.221)	0.998(0.582)	0.999(0.351)	0.336(0.06)
CC*	0.967(0.601)	1(0.858)	1(0.748)	0.73(0.35)
*I*/*σI*	5.88(1.32)	14.11(1.74)	13.51(0.44)	1.99(1.05)
Completeness [%]	93.7(77.8)	99.89(99.51)	98.56(81.91)	98.87(98.37)
Redundancy	7.4(2.6)	6.0(2.9)	10.2(36.6)	10.5(6.3)
	–	–	–	–
Refinement	–	–	–	–
Resolution [Å]	30.94–2.03(2.07–2.00)	22.24–1.50(1.53–1.50)	38.53–1.06(1.09–1.06)	39.14–1.7(1.83–1.7)
No. reflections	6871	17,701	51,068	13,634
			–	–
*R*_work_/*R*_free_	0.251/0.271(0.295/0.344)	0.200/0.223(0.273/0.274)	0.19/0.23(0.34/0.37)	0.2570/0.3080(0.290/0.291)
No. atoms	–	–		
Protein	863	897	863	849
Ligand/ion/Water	43	159	122	43
	–	–	–	–
*B*‐factors	–	–	–	–
Protein	25.01	14.53	13.41	24.95
Ligand/ion/Water	20.66	28.44	25.08	20.66
–	–	–	–	–
R.m.s. deviations	–	–	–	–
Bond Lengths [Å]	0.002	0.019	0.0125	0.0118
Bond angles [°]	0.45	1.48	2.231	2.1810
–	–	–	–	–
Ramachandran plot	–	–	–	–
Favored [%]	99.04	99.04	99.04	97.12
Allowed [%]	0.96	0.96	0.96	2.88
Disallowed [%]	0.00	0.00	0.00	0.00

#### 2.1.2 Overall Structural Consistency Assessed by Root Mean Square Deviation (RMSD) Superposition

**Figure 1:**
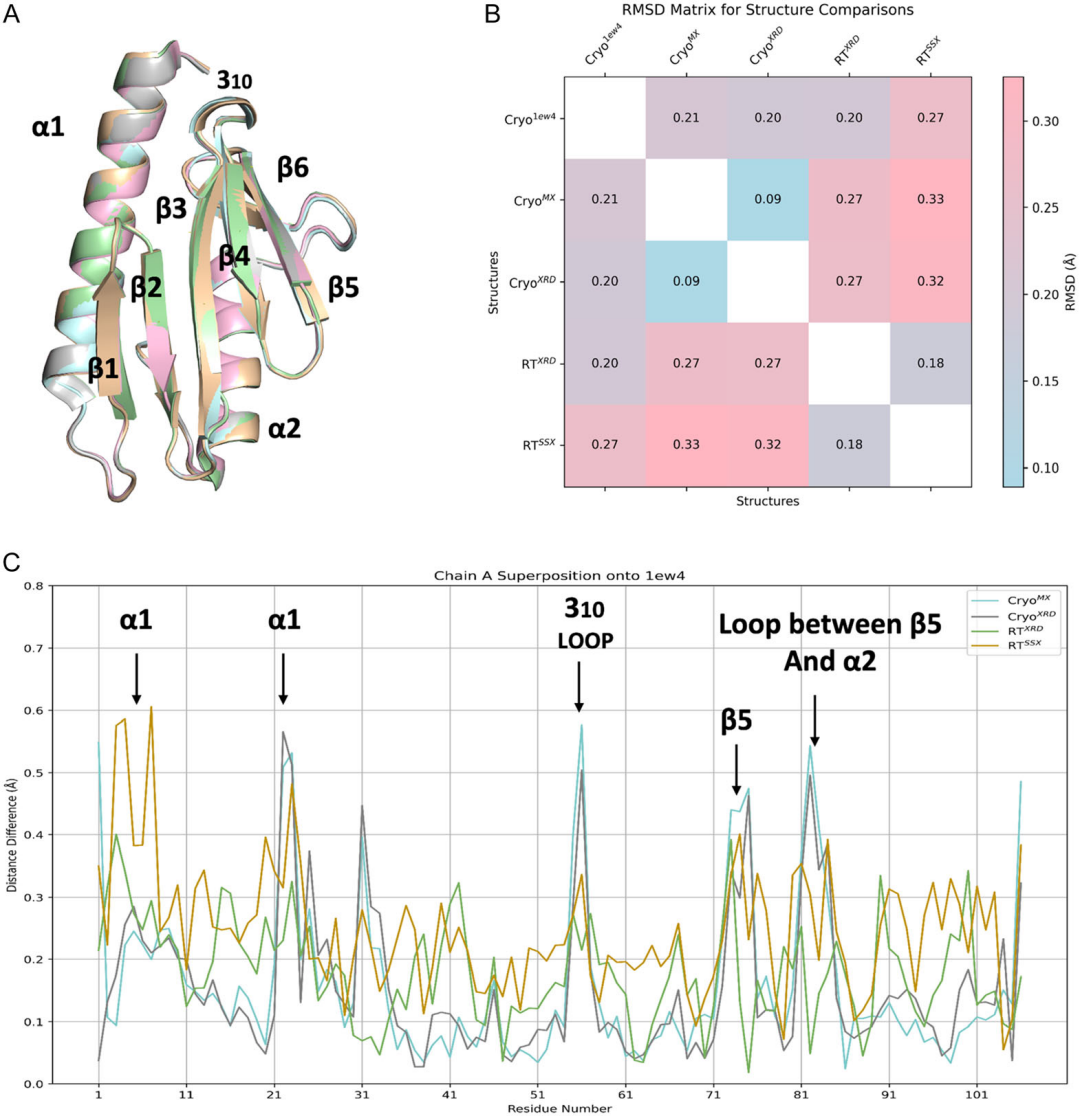
The superimposition of all structures onto the already published cryogenic structure (PDB:1ew4) A) pairwise RMSD matrix B). Pairwise alignment based on C*α* atoms reveals notable structural shifts when all four structures are superimposed on to the already published cryogenic structure (PDB:1ew4) C).

To evaluate structural variations among monomeric structures of CyaY obtained at different temperatures and the previously published cryogenic structure (PDB: 1ew4), each structure was superimposed **Figure** **1**A. The overall *β*‐sheet/*α*‐helix sandwich is preserved at all temperatures—CyaY remains a stable fold. The resulting Root Mean Square Deviation (RMSD) values ranged from 0.09 to 0.33 Å as shown in the heatmap matrix (Figure 1B), highlighting subtle conformational differences based on C*α* atom alignments. Lower values (closer to blue/white) indicate higher similarity; higher values (red) suggest more divergence. These findings indicate a moderate level of structural flexibility and heterogeneity among the individual structures. Pairwise alignments were conducted to examine positional variations of equivalent C*α* atoms, and the differences were mapped against the corresponding residue numbers (Figure 1C). Peaks in the plot represent regions with high structural variation. The flat regions (low RMSD) indicate conserved or stable regions like. Subtle shifts in certain loops and helices hint at regions of enhanced mobility when you go from cryogenic (<100 K) to near‐physiological (≈298 K) conditions. RMSD values between the two cryogenic structures were low (≈0.09–0.20 Å), indicating minimal structural variation at cryogenic temperatures. Comparisons between cryogenic and room‐temperature structures showed higher RMSD values (≈0.27–0.33 Å), reflecting a small yet consistent global conformational shift. The RMSD between the two room‐temperature structures was intermediate (≈0.18 Å), suggesting that the room‐temperature structures are more closely related to each other than to their cryogenic counterparts. Figure 1 collectively demonstrates that while the core structure of CyaY is highly conserved, certain regions exhibit flexibility or deviations across structures. These findings support the idea that cryocooling alters the conformational ensembles of side chains, affecting both solvent‐exposed and buried residues.^[^ ^20^ ^]^ The unit‐cell dimensions at room temperature are slightly larger than those under cryogenic conditions, expanding by approximately 1 Å in each dimension (Table 1). This expansion is likely due to increased thermal motion and lattice relaxation at ambient temperature. These structural shifts may have functional implications, such as in binding iron or partner proteins, offering targets for future mutagenesis or dynamics studies.

#### 2.1.3 Conserved and Variable Water Networks across Structures

**Figure 2:**
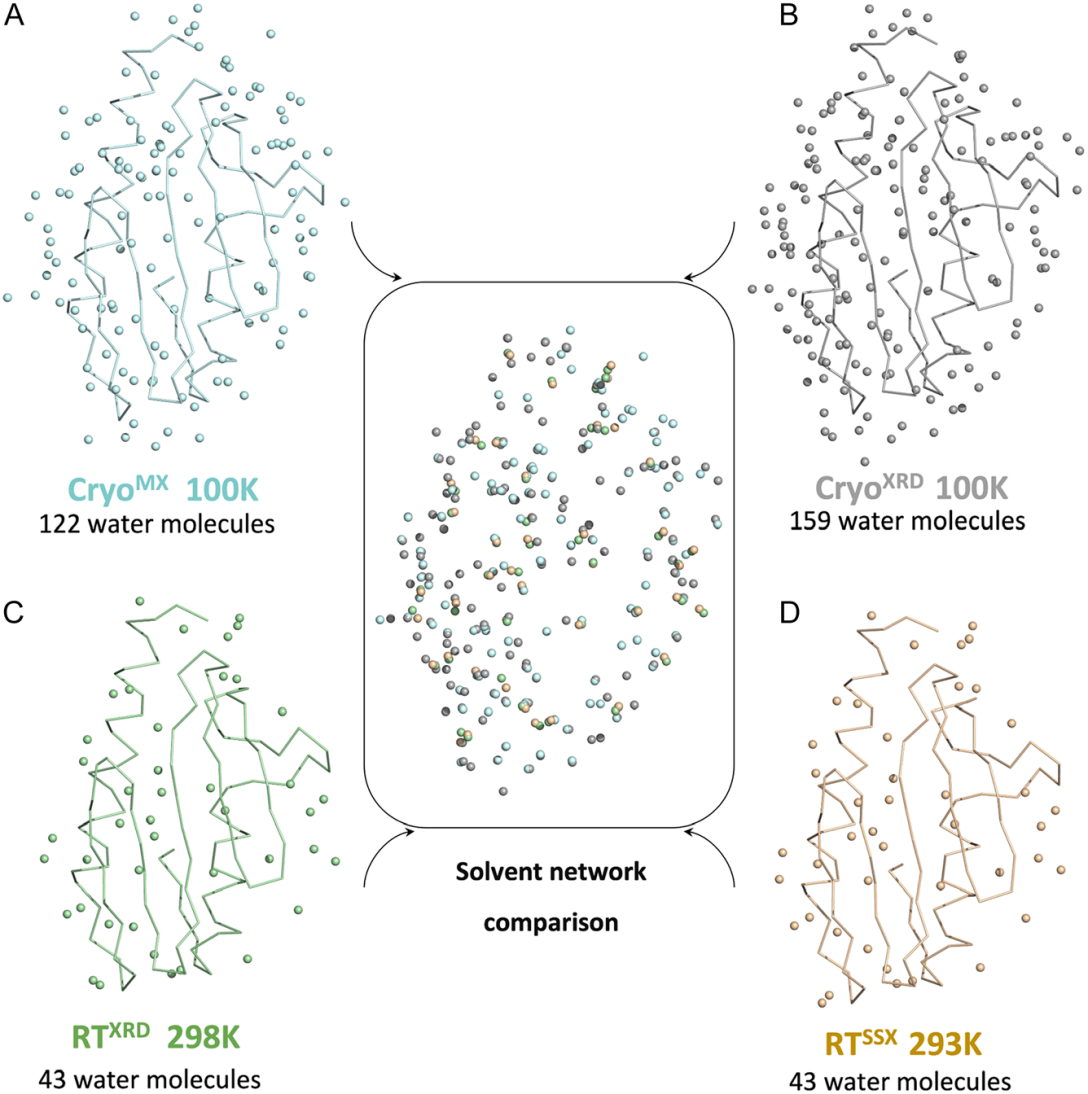
Comparison of water molecules across all our structures. Defined water molecules are shown on the Cryo^MX^ A), Cryo^XRD^ C), RT^XRD^ B), and RT^SSX^ D). Secondary structures are represented as lines, while water molecules are depicted as spheres. (Center) Superposition of water molecules from all four datasets highlights conserved and unique water positions across conditions.

To investigate how temperature influences solvation, we examined the water networks across all CyaY structures. This comparison revealed notable differences in the number and positioning of water molecules between room‐temperature and cryogenic conditions. Both room‐temperature structures revealed the same water network, while cryogenic structures contained more water molecules and exhibited variations in their arrangement (**Figure** **2**), as expected. Less water molecules in room temperature structures is likely because elevated temperatures increase the disorder of loosely coordinated water molecules; as has been previously observed by others.^[^ ^32^ ^,^ ^33^ ^]^ The presence of the same water molecules in room‐temperature structures suggests the formation of a stable hydration shell within the crystal lattice.^[^ ^34^ ^]^ However, cryogenic conditions can alter water positions or introduce additional molecules due to cryoprotectants and freezing effects.^[^ ^35^ ^]^ This underscores the importance of conducting crystallography under varying conditions, such as different temperatures, to map potential solvation patterns that may be critical for understanding the protein's behavior in diverse environments. Additionally, when we map ordered‐water positions onto the electrostatic surface (Supplementary Figure 1), we observe a roughly 20–30% reduction in tightly bound waters in room temperature structures compared to the cryogenic structures. While the electrostatic surface potential appears largely conserved across all structures, the loss of these waters leaves the acidic patches more exposed to the environment, which might affect how the protein interacts with ions, other proteins, or ligands.

**Figure 3:**
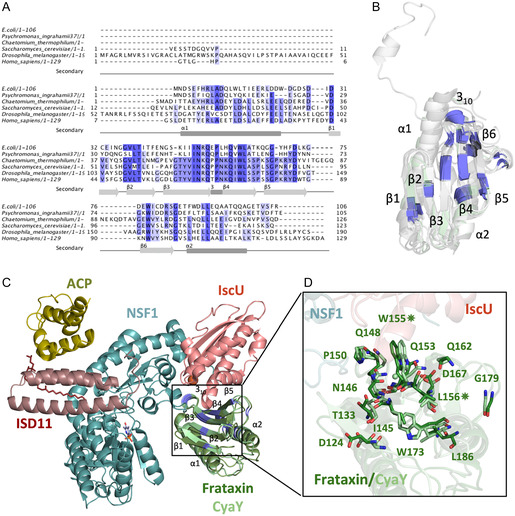
A) Multiple sequence alignment of frataxin homologs from various species including *E. coli* (PDB*:*1ew4), *P. ingrahamii* (PDB ID:4HS5), *C. thermophilum* (PDB ID:6FCO), *S. cerevisiae* (PDB ID:3OEQ), *D. melanogaster* (PDB ID:7N9I), and *H. sapiens* (PDB ID:3S4M). Conserved residues are highlighted in blue, and secondary structure elements (*α*‐helices and *β*‐strands) are annotated below the alignment. B) Highly conserved residues (darker blue) are shown in all superimposed structures based on sequence alignment results in panel A. The *E. coli* CyaY structure is depicted in pale green, while homologous structures are shown in varying shades of gray. C) The overall architecture of the eukaryotic Fe–S cluster assembly complex—comprising NFS1, ISD11, ACP, IscU, and frataxin—is shown based on the Cryo‐EM structure (PDB ID: 6NZU). The bacterial frataxin homolog, CyaY(PDB ID: 1EW4), has been superimposed onto the complex and is depicted in lighter green. D) Highly conserved residues are shown in stick representation, with residue numbering corresponding to the human eukaryotic frataxin sequence. The corresponding residues between human frataxin and *E. coli* CyaY are listed in Supplementary Table 1. Amino acids marked with asterisks indicate positions where point mutations are associated with severe cases of Friedreich's Ataxia.

Multiple sequence alignment revealed highly conserved residues, primarily within the *β*‐sheet core (*β*1–*β*6) of frataxin (**Figure** **3**A,B), suggesting their structural and functional importance. The strong conservation supports using *E. coli* CyaY as a simplified model to study human frataxin and Fe–S cluster biosynthesis.

**Figure 4:**
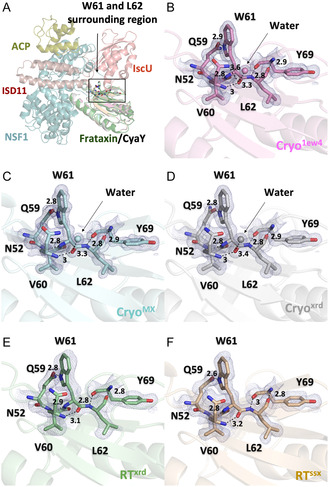
Structural environment surrounding W61 and L62 in E. coli CyaY at cryogenic and room temperature conditions—The eukaryotic Fe–S cluster assembly complex (PDB: 6NZU) is shown with bacterial frataxin homolog CyaY (PDB: 1EW4) superimposed in light green. The region surrounding W61 and L62 is highlighted in the box A) Electron density maps and hydrogen bonding networks are shown for five CyaY structures solved at cryogenic (Cryo^1EW4^, Cryo^MX^, Cryo^XRD^) and room temperature (RT^XRD^, RT^SSX^) B–F). Residues forming polar contacts with W61 and L62, along with nearby ordered water molecules, are shown with labeled distances in Ångstroms (Å). An ordered water molecule is consistently observed near W61 in all cryogenic structures but is absent in both room temperature datasets. While overall polar interactions are preserved across all structures, subtle variations in hydrogen bond distances are observed between cryogenic and room temperature structures.

As mentioned earlier, there are notable differences in the ordered water network and hydrogen bonding patterns across all structures. **Figure** **4** illustrates these interactions around the conserved residues W61 and L62, which correspond to W155 and L156 in human frataxin**.** These residues are located within a highly conserved region critical for interaction with IscU (Figure 3C,D), and mutations such as W155R and L156P in humans have been linked to Friedreich's Ataxia due to frataxin destabilization and impaired Fe–S cluster assembly.^[^ ^36^ ^]^ An ordered water molecule is consistently observed near W61 in all cryogenic structures, but is absent in the room temperature models. While polar contacts among neighboring residues are generally conserved in the cryogenic datasets, slight differences in interaction distances are observed when compared to the room temperature structures (Figure 4A–F).

The temperature‐dependent variability has direct implications for structure‐based drug design (SBDD), as hydration states influence binding pocket shape, polarity, and accessibility. Some water molecules may serve structural roles and should be preserved in docking simulations, while others may be displaceable, offering opportunities to design ligands that enhance binding through optimized interactions. These findings highlight the importance of incorporating structural flexibility and solvent dynamics into drug design strategies. Using CyaY as a model, combined with temperature‐resolved structural data, provides a valuable framework for identifying druggable sites and developing therapeutics for Friedreich's Ataxia.

#### 2.1.4 Temperature‐Dependent Variability in Key Side Chain Orientations

**Figure 5:**
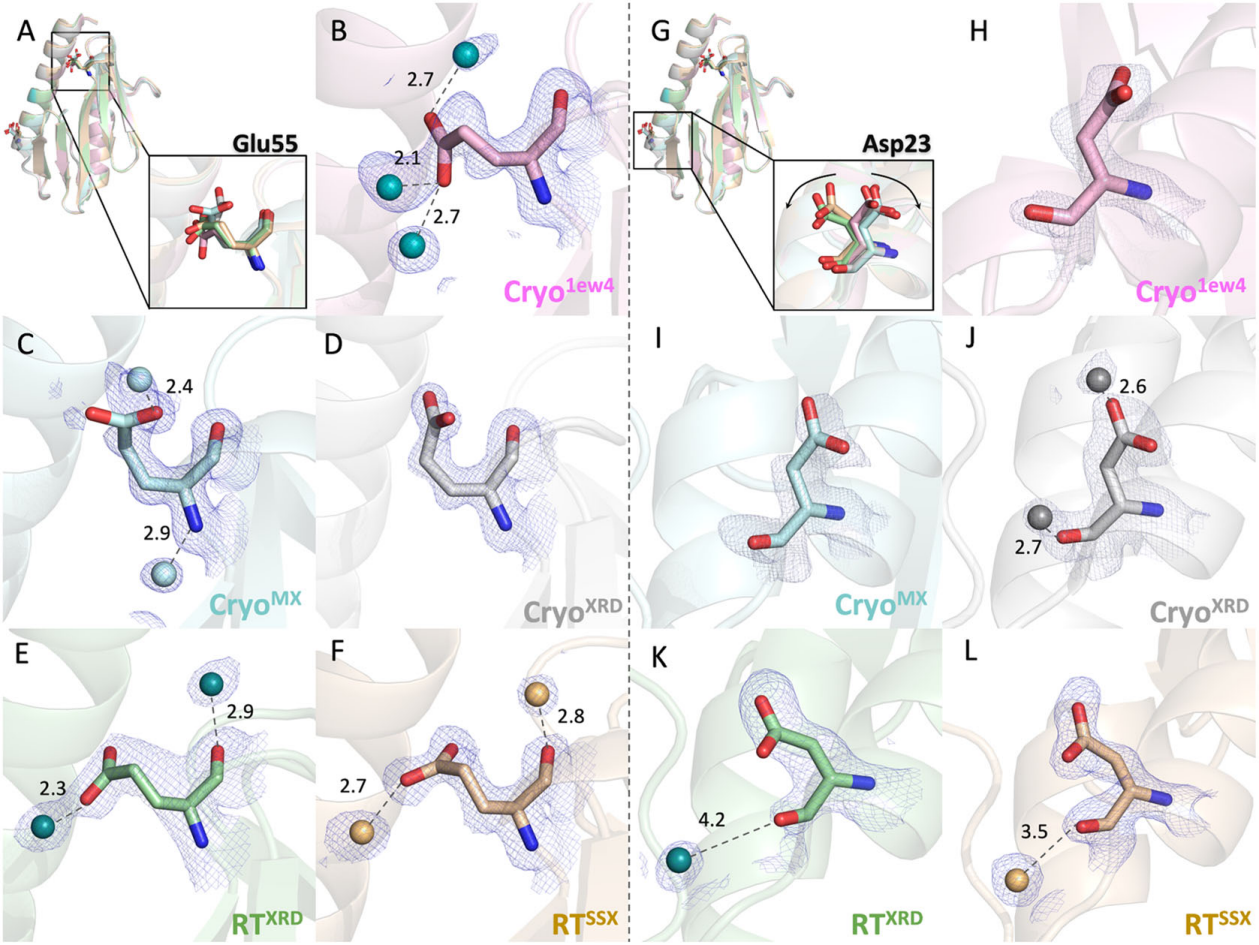
Side chain orientation comparison for Glu55 A–F) and Asp23 G–L) across all our structures. Superimposed structures are shown in A and G, highlighting the orientations of Glu55 and Asp23, respectively. Glu55 is shown in each structure from B to F, with water molecules in contact depicted as spheres. Asp 23 is shown in each structure from G to L, with water molecules in contact depicted as spheres. *2Fo‐Fc* simulated annealing‐omit map for all related residues are shown in tv‐blue. Figures are generated by PyMol version 2.3 (https://pymol.org/).

We compared all available structures to investigate potential conformational changes and observed variations in several key residues. According to NMR titration studies,^[^ ^37^ ^]^ Glu55 is located within the region of CyaY that interacts with IscU. In our analysis, the side chain orientation of Glu55 was consistent across the room‐temperature structures but differed in the cryogenic structures (**Figure** **5**A–F). These conformational differences were accompanied by shifts in the surrounding water molecules. Notably, in the room‐temperature models, the positions of water molecules near Glu55 remained conserved, maintaining nearly identical distances across both RT structures. We also observed differences in the side chain orientation of Asp23 across the structures. Asp23 is an important residue due to its direct involvement in iron binding and its role in interacting with IscS. In our comparisons, the side chain orientation of Asp23 was consistent in the room‐temperature structures but varied in the cryogenic ones. Similarly, the surrounding water molecules in the room‐temperature models occupied conserved positions and maintained nearly identical distances (Figure 5G–L).

#### 2.1.5 Visualization of Atomic Flexibility via Thermal Ellipsoid Representation

**Figure 6:**
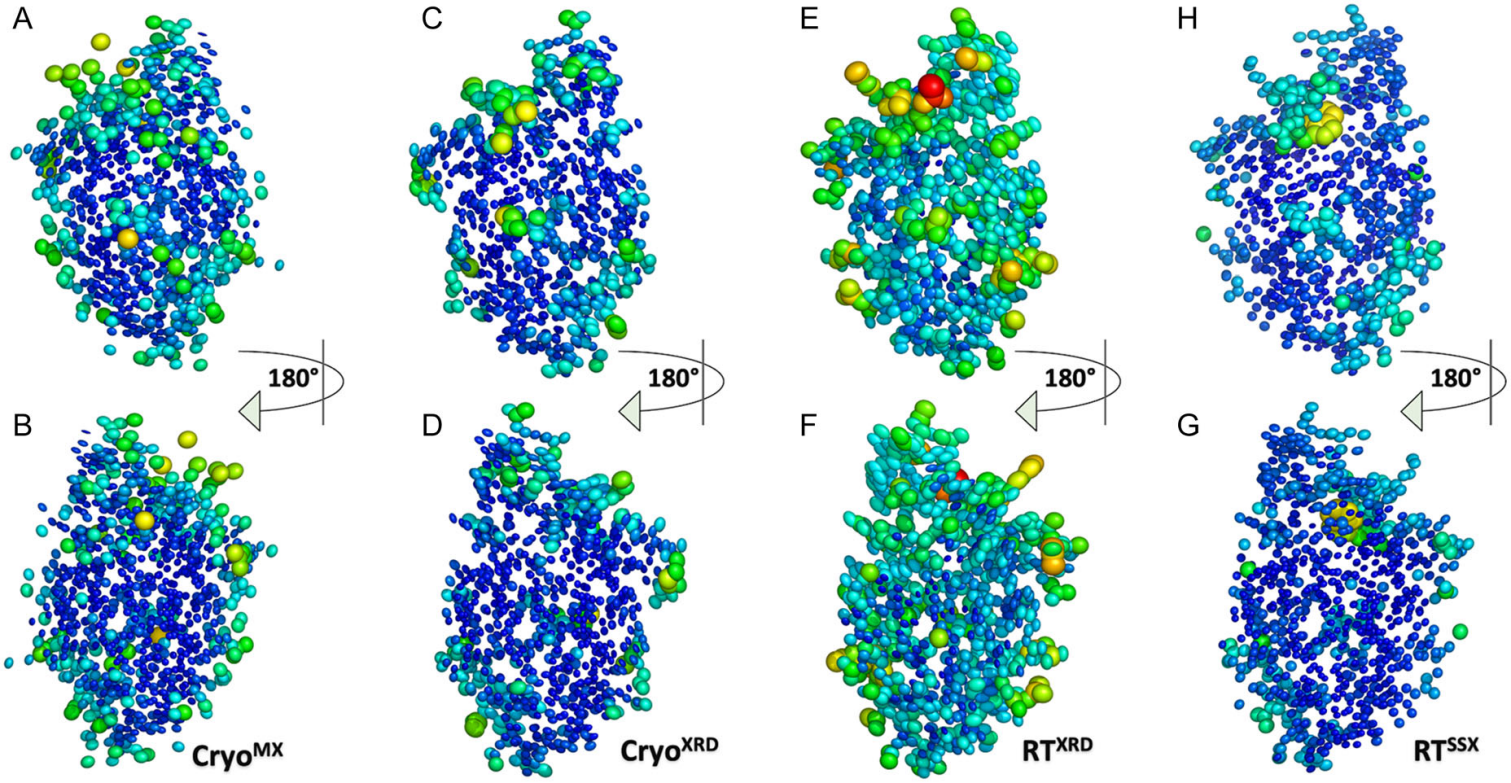
Representation of thermal ellipsoid structures of cryo^MX^ A), cryo^XRD^ B), RT^XRD^ E), RT^SSX^ H). The corresponding structures in the bottom row B–G) are shown rotated 180° around the *X*‐axis for better visualization. Figures are generated by PyMol version 2.3 (https://pymol.org/).

We then conducted a comparative analysis of all structures using thermal ellipsoid models. Ellipsoid models were generated from temperature factors (B‐factors) using PyMOL to visually emphasize differences in atomic flexibility. These are clearly depicted in the thermal ellipsoid representations, with colors ranging from blue (least flexible) to red (most flexible), corresponding to B‐factor values between 3.34 and 96.18 (**Figure** **6**). Our analysis revealed that both cryogenic structures exhibit compact, uniformly sized ellipsoids predominantly colored in blue and green, indicating consistently low atomic flexibility throughout the protein (Figure 6A–D).These structures show generally lower B‐factors and usually have fewer regions of high flexibility (Supplementary Figure 2A,B). The RT^XRD^ structure exhibited larger, more elongated thermal ellipsoids, reflecting increased atomic vibrations and greater dynamic flexibility compared to the other structures (Figure 6E,F). RT structures exhibit generally higher B‐factors (Supplementary Figure 2C,D). The RT^SSX^ still shows elevated flexibility (larger ellipsoids than cryo structures), but less extreme than RT^XRD^ (Figure 6H,G). These differences are essential when considering biological relevance—flexibility at RT temperature may better represent native physiological conditions.

### 2.2 Improved Electron Density of a Key Residue in Room‐Temperature Structures, Unresolved in Cryogenic Data so Far

**Figure 7:**
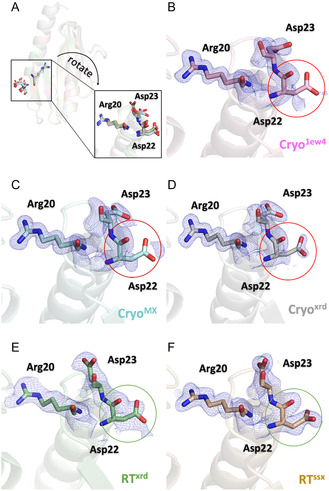
An example of a key residue exhibiting a higher degree of order in room‐temperature structures compared to cryogenic structures is shown. A) A zoomed‐out view providing a general overview is shown in panel A. The 2FoFc electron density maps for the Arg20‐Asp22‐Asp23 residues are displayed in all structures B–F), contoured at 1*σ* (tv‐blue).

One notable temperature‐related difference in the structures of CyaY is that certain residues are better defined in the room‐temperature (RT) models. This suggests that RT data may provide more structural information on regions that are typically less ordered. In our RT structures, we were able to confidently model the key residue Asp22 (**Figure** **7**E,F), whereas this loop is only partially resolved in both our cryogenic structures and the previously published cryogenic structure (PDB:1ew4) (Figure 7B–D). Interestingly, Asp22 directly interacts with iron and is also involved in binding to IscS, as indicated by NMR titration studies.^[^ ^37^ ^]^

## 3 Conclusion

Compared to cryogenic methods, room‐temperature data collection captures functionally relevant conformational flexibility, allowing access to catalytic processes, photocycles, and interaction‐dependent binding modes that are often suppressed at lower temperatures.^[^ ^38^ ^]^ Fixed‐target serial crystallography enables high‐throughput collection of diffraction data from small crystals at room temperature, making it especially valuable for challenging samples that are sensitive to radiation damage or incompatible with cryoprotection.^[^ ^28^ ^]^

A major limitation of both conventional cryogenic X‐ray crystallography and modern cryo‐electron microscopy (cryo‐EM) is that they only capture structural information of protein complexes in a frozen state. In contrast, serial crystallography is an emerging method that allows for the study of protein structures at room temperature.

Several studies have revealed interesting structural differences related to temperature by examining the conformational landscape of proteins related to temperature. For example, multi‐temperature X‐ray crystallography has been used to explore the temperature dependent dynamics of the SARS‐CoV‐2 main protease (Mpro), uncovering unexpected structural dynamics and offering new insights relevant to drug design.^[^ ^29^ ^]^ In another study, an emerging approach involving room temperature (RT) data collection was applied to soluble epoxide hydrolase. A comparison between RT structures and previously reported cryogenic‐temperature structures revealed temperature‐dependent variations in ligand‐binding modes. Notably, flexible loops were more clearly resolved at RT, emphasizing the potential benefits of studying protein dynamics under more physiologically relevant conditions.^[^ ^39^ ^]^

This study highlights both the current challenges and the potential future advancements of serial crystallography, particularly in its application to pharmaceutical drug discovery.

While sulfur transfer has been somewhat characterized in the Fe‐S cluster biogenesis system,^[^ ^40^, ^41^ ^–^ ^42^ ^]^ the incorporation of iron into the Fe‐S cluster is still an unclear process from a structural perspective. Proteins involved in iron–sulfur cluster biogenesis often coordinate metal ions and are therefore highly susceptible to radiation damage during conventional cryogenic X‐ray experiments. Room‐temperature structures—especially those obtained by serial synchrotron crystallography (SSX)—provide an essential foundation for more advanced studies. Serial femtosecond crystallography (SFX) at X‐ray free‐electron lasers (XFELs) offers a powerful solution.^[^ ^43^ ^]^ Building on SSX datasets, SFX experiments can be designed to explore the real‐time dynamics of ISC assembly under near‐physiological conditions.

In several ataxia treatment studies, frataxin itself has emerged as a key drug target.^[^ ^44^ ^]^ Combining cryogenic and room‐temperature structures CyaY—the bacterial homolog of frataxin—enhances our drug‐design efforts by revealing dynamic side‐chain rotamers, and key water‐mediated interactions that cryo alone may miss. Ensemble docking against these structures—and explicit consideration of conserved versus displaceable waters—allows us to improve ligand affinity and specificity under physiological conditions.

## 4 Experimental Section

4.1

4.1.1

##### Protein Expression and Purification Preparation

The pET28a+ plasmid containing N‐terminal hexahistidine‐Sumo‐tagged CyaY gene was transformed into and overexpressed in *E. coli* RosettaTM BL21 (DE3) strain. The cells were grown at 37 °C in 4L LB media supplemented by 50 µl ml^–1^ kanamycin and 35 µl ml^–1^ chloramphenicol for large‐scale protein expression until OD_600_ reached the 0.6–0.8 range. Then 1:1000 (v:v) 0.4 mM Isopropyl *β*‐D‐1‐thiogalactopyranoside (IPTG) was added at 0.4 µM final concentration and overexpression continued at 18 °C for 18 h. Cells were harvested at 3500 rpm by Beckman Allegra 15R Desktop Centrifuge and resuspended in a lysis buffer containing 20 mM Tris–HCl (pH 7.5), 500 mM NaCl, 30 mM imidazole, and 5 mM *β*‐mercaptoethanol. Then, cells were lysed by sonication by using a Branson W250 sonicator (Brookfield, CT, USA) followed by ultracentrifugation at 35,000 rpm at Beckman Optima L‐80XP ultracentrifuge equipped with Ti‐45 rotor to isolate the soluble supernatant extract. The affinity chromatography purification was performed using the Ni‐NTA affinity column (Qiagen, Venlo, Netherlands), and the purified CyaY protein was then placed in the dialysis buffer and treated by Ulp1 to remove the N‐terminal hexahistidine‐Sumo tag. Cleaved CyaY was separated from uncleaved protein by passing the protein mixture through the Ni‐NTA affinity column, and the flowthrough, containing pure cleaved CyaY protein, was collected. The cleaved native CyaY protein was further concentrated to a final concentration of 3 mg ml^–1^ using (Amicon 10 KDa MWCO) ultrafiltration columns and it was kept at −80 for future experimentation.

##### Crystallization

Sitting drop vapor diffusion microbatch under oil crystallization technique was used for crystallization of CyaY protein at 4 °C. Search for crystallization conditions was performed by adding purified CyaY to the commercial sparse matrix and grid crystal screen conditions in the 72 well Terasaki crystallization plates with the 0.83 µl protein to cocktail ratio of 1:1 (v/v), subsequently covered with 16.6 µl paraffin oil (Tekkim Kimya, Istanbul, Türkiye). Approximately 3000 commercial sparse matrix and grid screen crystallization conditions were set up and examined.^[^ ^45^ ^]^ CyaY crystals were obtained using crystallization conditions (Grid‐NaCl) containing 4 M NaCl, 0.1 M citric acid, pH:4 within two weeks. Crystallization conditions were provided by Hampton Research, USA. Since we identified a condition that produces large, well‐diffracting crystals, it was selected for further optimization to generate microcrystals for SSX data collection. To prepare microcrystals of CyaY protein using seeding, we started by crushing larger crystals into smaller fragments to create a concentrated seed stock. Crystals were identified under a microscope, and using a probe, they were carefully crushed within the drop well. The crushed crystal slurry was transferred into a Seed Bead tube containing crystallization solution, then vortexed on ice to ensure uniformity. This seed stock was used in Microseed Matrix Screening (MMS), where it was mixed with protein and reservoir solutions to promote the growth of microcrystals under optimized conditions. The process allowed for the consistent generation of high‐quality microcrystals suitable for serial crystallography.

##### Room Temperature Data Collection at XRD and Processing and Refinement

Ambient temperature X‐ray crystallographic data was collected using Rigaku's XtaLAB Synergy R Flow XRD system, as outlined in Gul et al. 2022.^[^ ^46^ ^]^ Multiple crystals were screened using the modified adapter of the *XtalCheck‐S* plate reader. After selecting well‐diffracting crystals, diffraction data were collected from multiple crystals. The duration of exposure time was optimized to minimize the potential damage caused by radiation. A total of 3 crystals were used to collect complete diffraction data. Diffraction data were collected for 20 min (631 frames total). The detector distance was set at 91.00 mm, while the scan width was 0.50° and the exposure time was 2.00 s per image. The diffraction data were set up in *CrysAlisPro* to complete the automated data collection. The collected data was then merged using *proffit* merge process with *CrysAlisPro* 1.171.42.59a software (Rigaku OxfordDiffraction, 2022) to produce an integrated reflection dataset (*.mtz) file for further analysis, as described in Gul et al. 2022.^[^ ^46^ ^]^ The structure was determined by using molecular replacement with the *PHASER‐MR* program implemented in the *PHENIX suite*,^[^ ^47^ ^]^ using the cryogenic CyaY crystal structure (PDB code: 1EW4) as the starting search model.^[^ ^47^ ^]^ Following rigid body and simulated annealing refinement, individual coordinates and TLS parameters were refined. Potential positions of altered side chains and water molecules were examined, and the models were constructed/reconstructed using the COOT program.^[^ ^48^ ^]^ Final structure refinement was performed using phenix.refine in *PHENIX*.^[^ ^48^ ^]^ Structural figures were generated using *PyMOL* (Schrödinger, LLC)(https://pymol.org/).

##### Cryogenic Temperature Data Collection at XRD and Processing and Refinement

Cryogenic temperature X‐ray crystallographic data were collected using Rigaku's XtaLAB Synergy R Flow XRD system, as outlined in Atalay et al. 2022.^[^ ^49^ ^]^ CyaY crystals were flash‐frozen in liquid nitrogen before being loaded onto a cryocooled sample puck (catalog No. M‐CP‐111−021, MiTeGen, USA). The loaded puck was then transferred and located within the sample dewar at the Turkish Light Source^[^ ^49^ ^]^ for screening and data collection. The sample was kept at low temperatures during data collection using Oxford Cryosystems's Cryostream 800 plus which was set to a temperature of 100K. The PhotonJet‐R X‐ray generator was utilized at 40 kV, 30 mA, and 1200.0 W, producing a beam intensity of 10%. Diffraction data were collected for 12 min (362 frames total). The detector distance was set at 60.11 mm, while the scan width was 0.50° and the exposure time was 2.00 s per image. Collected data was processed and scaled using *CrysAlisPro* 1.171.42.59a software (Rigaku Oxford Diffraction, 2022), and then finalized to allow removal of outliers and modification of the space group. Finally, the processed data were exported to *.mtz format. Molecular replacement and refinement were carried out exactly as described in the Room‐Temperature Data Collection section. Structural figures were generated using *PyMOL* (Schrödinger, LLC)(https://pymol.org/).

##### Serial Synchrotron Crystallography Data Collection and Processing and Refinement

CyaY Serial synchrotron crystallography (SSX) data were collected at the P14 beamline, operated by EMBL at PETRA III, DESY, Hamburg, Germany.^[^ ^50^ ^]^ Crystals were mounted on silicon chips and scanned at room temperature using a 12.7 keV beam (10 µm diameter, 1.2 × 10^12^ photons s^−^^1^) with 5 ms exposures on an EIGER 4 M detector. Diffraction images were processed (indexing, integration, merging, MTZ conversion) in CrystFEL 0.10.1,^[^ ^51^ ^]^ then truncated, phased, and refined in CCP4 Cloud.^[^ ^52^ ^]^ Molecular replacement used CyaY (PDB 1EW4) in Phaser, followed by rigid‐body and restrained refinement in REFMAC5.^[^ ^53^ ^]^ Model building was done in Coot, and figures were prepared in *PyMOL*(Schrödinger, LLC)(https://pymol.org/).

##### Cryogenic Temperature Data Collection at Diamond and Processing and Refinement

For X‐ray diffraction, crystals were mounted using a 0.3–0.4 µm nylon loop and immediately flash‐frozen in liquid nitrogen. Diffraction experiments were carried out at the Diamond Light Source on the macromolecular crystallography beamline I04 (Harwell, UK), using a wavelength of 0.9795 Å. Data were collected over a 360° rotation of the omega axis with an increment of 0.1° per image. The diffraction data were processed using *autoPROC* ^[^ ^54^ ^]^ Structure determination was performed via molecular replacement using *PHASER* (from the CCP4 cloud), with coordinates from the E.coli CyaY structure (PDB ID: 1EW4) as the research model. Model building and manual adjustment were carried out in *Coot*, and final refinement was completed using *PHENIX*. Figures were prepared in *PyMOL*(Schrödinger, LLC)(https://pymol.org/).^[^ ^55^, ^56^, ^57^, ^58^ ^–^ ^59^ ^]^

## Conflict of Interest

The authors declare no conflict of interest.

## Author Contributions

**Alaleh Shafiei**: conceptualization (lead); data curation (lead); formal analysis (lead); investigation (lead); methodology (lead); project administration (lead); software (lead); validation (lead); visualization (lead); writing—original draft (lead); writing—review & editing (lead). **Nilufer Baldir**: writing—review & editing (supporting). **Jongbum Na**: writing—review & editing (supporting). **Jin Hae Kim**: conceptualization (equal); funding acquisition (equal); investigation (supporting); methodology (supporting); project administration (supporting); resources (equal); supervision (supporting); visualization (supporting); writing—review & editing (supporting). **Hasan DeMirci**: conceptualization (equal); data curation (supporting); formal analysis (supporting); funding acquisition (equal); investigation (equal); methodology (equal); project administration (equal); resources (lead); software (supporting); supervision (lead); validation (equal); visualization (lead); writing—review & editing (supporting).

## Supporting information

Supplementary Material

## Data Availability

The data that support the findings of this study are available in the supplementary material of this article.
