# Supplementary material for: Comparative Structural Analysis of Escherichia Coli Cyay at Room and Cryogenic Temperatures Using Macromolecular and Serial Crystallography
Source: Chembiochem. 2025 Oct 28;26(20):e202500442. doi: 10.1002/cbic.202500442 (PMC12582159; doi:10.1002/cbic.202500442)
Supplement: Supplementary file 1 — Supplementary Material [file CBIC-26-e202500442-s001.pdf]

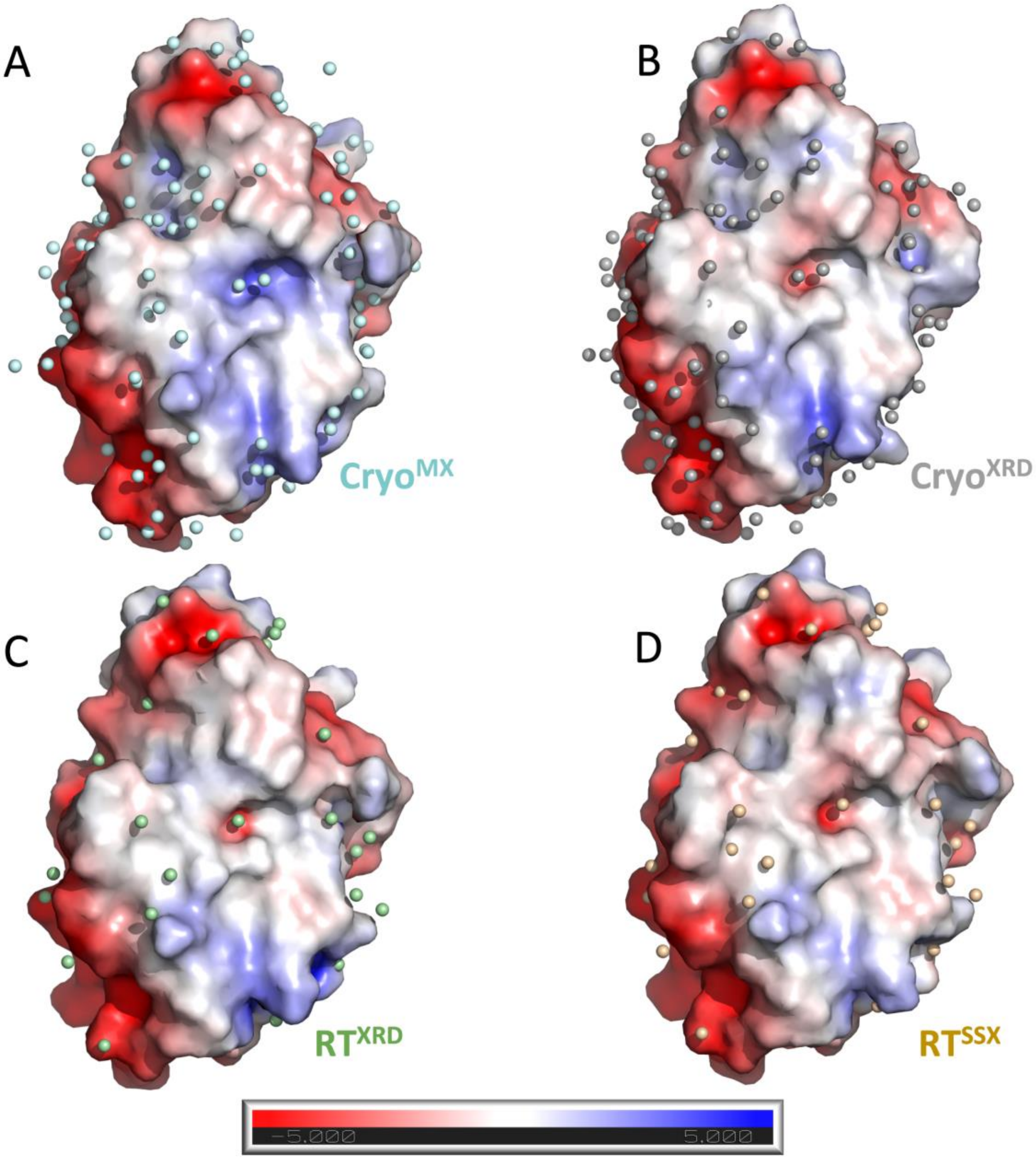

Supplementary figure 1. A comparison of water molecules across all structures is shown along with electrostatic surface representations. Defined water molecules are displayed on the surfaces of $Cryo^{MX}$ (A), Cryo ^{XRD} (C), RT $_{XRD}$ (B), and RT ^{SSX} (D). Electrostatic surface potentials were calculated using APBS in PyMol, with each structure represented by a color spectrum, where red indicates the lowest electrostatic potential energy and blue the highest.

Frataxin	CyaY	Location

D124	D31	β1
T133	T40	β2
I145	I51	β3
N146	N52	β3
Q148	Q54	β3
P150	P56	loop 3_{10}
Q153	Q59	β4
W155✷	W61	β4
L156✷	L62	β4
G162	G67	loop
D167	D72	β5
W173	W78	β6
G179	G84	loop
L186	L91	α2

Supplementary Table 1: Corresponding highly conserved residues between human frataxin and *E. coli* CyaY based on the multiple sequence alignment shown in Figure X. Residue numbering corresponds to the human frataxin sequence and the *E. coli* CyaY sequence, respectively. Amino acids marked with asterisks indicate positions where point mutations are associated with severe cases of Friedreich's Ataxia.

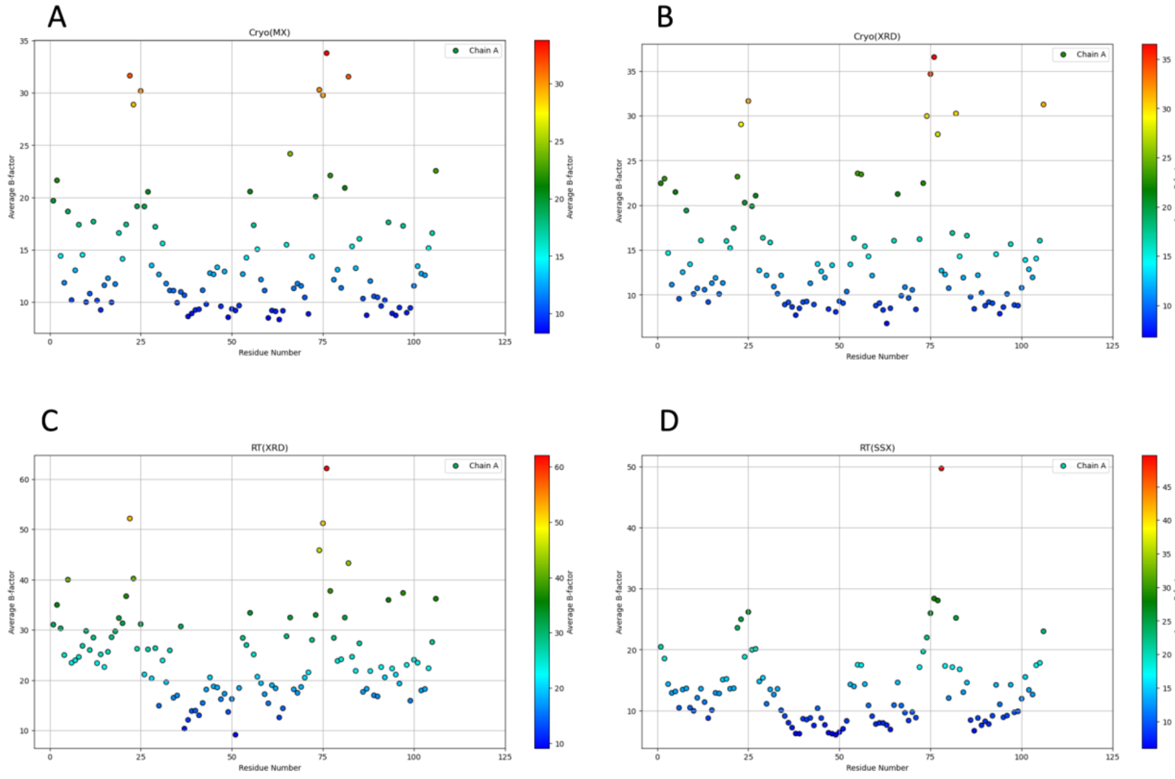

Supplementary figure 2. Comparison of residue-specific average B-factors across protein structures solved at cryogenic ($Cryo^{MX}$, $Cryo^{XRD}$) and room temperature (RT^{XRD}, RT^{SSX}) conditions**.**
